# Variability of Prefrontal Neuronal Discharges before and after Training in a Working Memory Task

**DOI:** 10.1371/journal.pone.0041053

**Published:** 2012-07-25

**Authors:** Xue-Lian Qi, Christos Constantinidis

**Affiliations:** Department of Neurobiology and Anatomy, Wake Forest University School of Medicine, Winston-Salem, North Carolina, United States of America; Heidelberg University, Germany

## Abstract

Variability of neural discharges can be revealing about the computations and network properties of neuronal populations during the performance of cognitive tasks. We sought to quantify neuronal variability in the prefrontal cortex of naïve monkeys that were only required to fixate, and to examine how this measure was altered by learning and execution of a working memory task. We therefore performed analysis of a large database of recordings in the same animals, using the same stimuli, before and after training. Our results indicate that the Fano Factor, a measure of variability, differs across neurons depending on their functional properties both before and after learning. Fano Factor generally decreased after learning the task. Variability was modulated by task events and displayed lowest values during the stimulus presentation. Nonetheless, the decrease in variability after training was present even prior to the presentation of any stimuli, in the fixation period. The greatest decreases were observed comparing populations of neurons that exhibited elevated firing rate during the trial events. Our results offer insights on how properties of the prefrontal network are affected by performance of a cognitive task.

## Introduction

Discharge rates of cortical neurons are highly variable from trial to trial during the presentation of identical sensory stimuli or execution of motor movements to the same targets, a phenomenon that is insightful about the organization of neural circuits [1], [2]. Furthermore, trial-to-trial variability is weakly but significantly correlated across neurons [3], [4], [5], [6], [7], although the precise degree of this correlation is a matter of debate [8]. This variability and correlation has important implications about the nature of information encoding in neuronal populations [9]. Obtaining experimental measures of variability has therefore been very valuable for the insight it offers on neuronal networks [10].

More recently, it has been recognized that variability can be an important indicator of neuronal computations and cognitive states. Variability of individual neuronal firing rates to stimuli was shown to decrease when attention was directed to them [11] and during the presentation of a sensory stimulus, presumably as a result of bottom-up attention [12]. A similar decrease in variability has been reported in preparation of a motor movement [13]. Conversely, the variance of firing rate was shown to increase in the course of decision making, and as a function of the number of alternative choices [14]. The correlation between the discharges of a pair of neurons also varies dynamically depending on whether the two neurons are pooled together for the same computation [15], [16]. These results indicate that variability and correlation are not fixed properties of neuronal networks but they vary dynamically as a result of cognitive operations.

Virtually all estimates of variability in areas of the association cortex have been obtained in animals trained to perform a behavioral task [10]. In view of the recent findings suggesting modulation of variability by cognitive factors, the reported values on which models of neuronal organization have been based seem likely to have been influenced by the cognitive operations imposed by the task. We were therefore motivated to analyze neuronal recordings in the prefrontal cortex of monkeys before they had been trained to perform a task and were simply required to fixate. We compared these with recordings obtained from the same animals after they were trained to perform a working memory task [17], [18]. Importantly, these experiments used identical stimuli presented with the same timing. We were therefore able to quantify the variability of the prefrontal network absent the effect of mental operations dictated by the task, and to reveal the nature of changes on this variable in the same animals as a result of learning to perform a working memory task.

## Results

**Figure 1:**
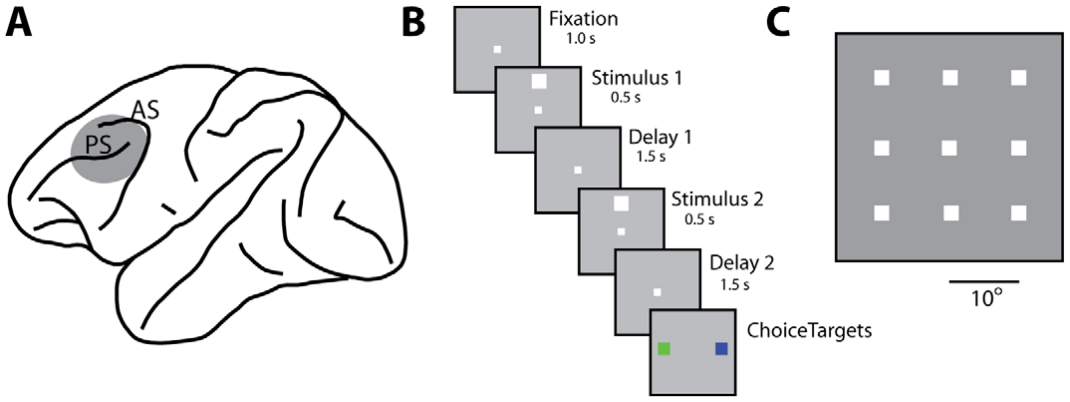
Brain areas, task, and stimuli. A) Schematic diagram of the monkey brain with the area of recordings highlighted. Abbreviations: AS, Arcuate Sulcus; PS, Principal Sulcus. B) Successive frames illustrate the sequence of stimulus presentations. In the pre-training stage the animals were rewarded for maintaining fixation after the end of the second delay period. After training, the animals were presented with two choice targets and were required to saccade to a green target if the two stimuli were matching and to a blue target otherwise. C) Stimulus set used for the analysis presented in this paper. Stimuli were white squares presented on a 3×3 grid, with a spacing of 10° from each other.

We analyzed neuronal activity from the prefrontal cortex (Fig. 1A) of three monkeys, recorded before and after training in a spatial working memory task [18]. After training, monkeys were required to view two stimuli presented in sequence and to determine whether they appeared at the same location or not. The monkeys indicated the match or nonmatch status of the stimuli by making an eye movement to a green or blue choice target that appeared after the presentation of the stimuli (Fig. 1B). Prior to training, the animals viewed the exact same stimuli, presented with an identical time course but no choice targets appeared at the end of the trial, and the monkeys were rewarded merely for maintaining fixation. We analyze here responses to the spatial set of stimuli, which involved white squares appearing at one of nine possible locations, spaced by 10° apart (Fig. 1C).

Our analysis drew from a total of 1324 neurons recorded prior to training and 1351 neurons recorded after training in the working memory task (Fig. 1B–C). Prior to training, 565 (43%) of these neurons responded to any aspect of the task with a significant change in firing rate over the baseline, 315 neurons (24%) responded to the visual stimuli with a significant elevation of firing rate and 232 neurons (18%) exhibited significantly elevated delay period activity. After training, 760 (56%) responded to any aspect of the task, 425 neurons responded to the stimuli (31%) and 449 exhibited delay period activity (33%).

### Variability across the time course of a trial

**Figure 2:**
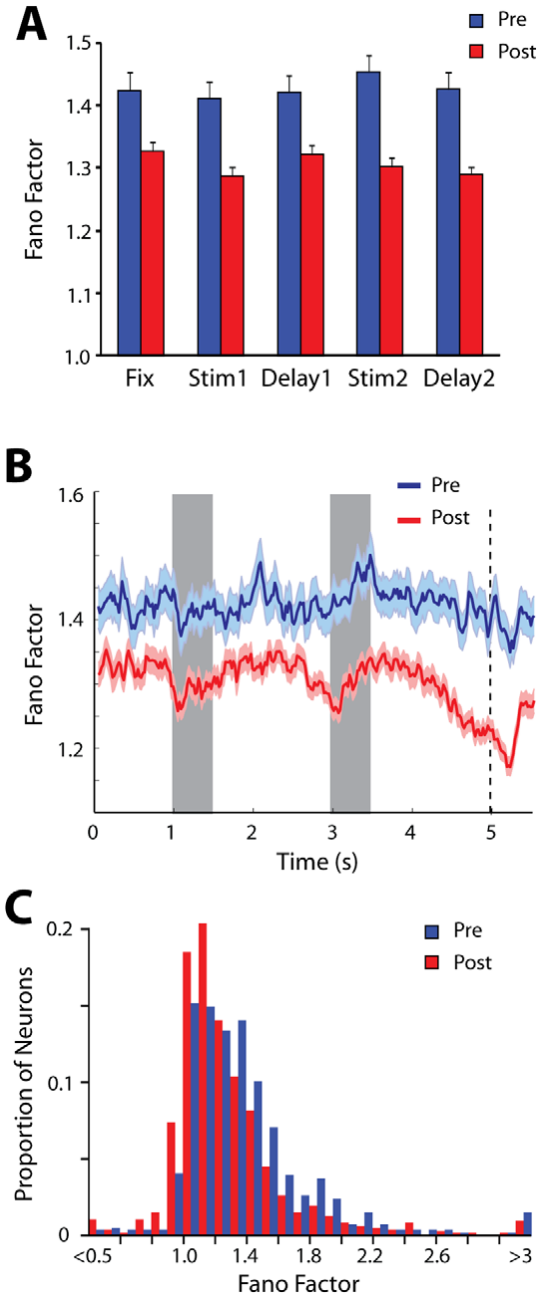
Average Fano Factor. A) Average Fano Factor values during the fixation period, stimulus presentation period and delay period for all neurons with significant responses to the stimuli (N = 518 pre; 741 post-training). Error bars represent the standard error of the mean. B) Time course of Fano Factor in 100 ms successive bins. Shaded area around each curve represents the standard error of the mean. Gray bars in the plot represent the time of stimulus presentation; vertical line, the time of reward in the pre-training condition and the time of choice targets appearance in the after training condition. C) Distribution of Fano Factor values computed during the stimulus period, for all neurons before and after training.

We initially sought to characterize the variance of neuronal responses in monkeys naïve to training. For this analysis we relied on the Fano Factor (variance divided by mean) of neuronal firing rates. The mean value across neuronal responses was *FF*  = 1.42 for the sample of neurons that responded to stimuli (Fig. 2A, blue bars). Average Fano Factor values obtained in each of the task epochs appeared to vary little between epochs, although a time-resolved calculation of the Fano Factor revealed a transient decrease around the time of the first stimulus presentation (Fig. 2B, blue line). This drop in variability was consistent with stimulus effects previously reported in multiple cortical areas [12]. We obtained a wide range of Fano Factor values across prefrontal neurons though approximately 70% of the neurons fell in the range of 1.0 and 1.5 (Fig. 2C, blue bars).

Recordings obtained after training (Fig. 2A) revealed a significant decrease in Fano Factor values (2-way ANOVA, p<10^−5^). The mean value across neuronal responses was *FF*  = 1.31 for neurons that responded to stimuli (Fig. 2A, red bars). This represented an overall decrease of 7.5% compared to before training, which was in the same scale as the decrease induced by the stimulus (5.2% decrease for the lowest stimulus-period bin compared to the average value across the fixation period, for the post-training data). The results indicate that performing the task exerts a powerful modulation on neuronal variability.

As was the case for data collected before training, Fano Factor values varied considerably on a neuron by neuron basis (Fig. 2C). The pre- and post-training distributions exhibited near identical kurtosis (1.72 and 1.73 respectively), suggesting that the entire distribution of Fano Factor values had shifted laterally, to lower values after training.

Although Fano Factor values decreased in all task epochs, the time course of the Fano Factor after training displayed a complex pattern, somewhat similar to what has been described in other tasks [19]. We observed decreases around each of the two stimulus presentations, as well as a large decrease in variability after the second stimulus had been presented and prior to the appearance of the choice targets, which required a judgment about the matching or not status of the stimuli (Fig. 2B). Average Fano Factor values at the last 500 ms of the second delay period represented a 6.7% decrease compared to the fixation period. This temporal structure was largely absent prior to training.

### Influence of firing rate

**Figure 3:**
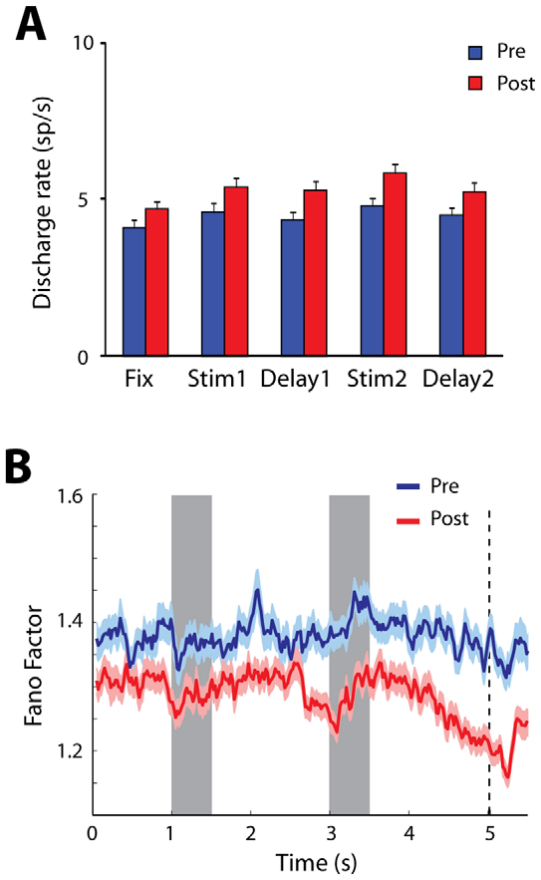
Influence of discharge rate . A) Mean discharge rate across all stimulus conditions for neurons with stimulus responses, recorded before and after training. B). Time of course of Fano Factor for neuron samples recorded before and after training, matched for mean discharge rate. Conventions are the same as in Figure 2B.

Although Fano Factor scales variance by mean discharge rate, neurons firing at higher rates tend to exhibit less variability than neurons firing at lower rates [11]. Firing rates after training tended to be higher overall [17], [18], and this was the case for the sample we used for the Fano Factor analysis (Fig. 3A). It is possible therefore that the decreases in Fano Factor we observed were caused entirely by changes in mean firing rates. To account for this possibility, we computed Fano Factor values from subsets of neurons recorded before and after training that were matched for firing rate in the stimulus presentation period (see Methods). Fano Factor in this rate-matched sample was 6.7% lower after training, compared to 7.5% for the entire sample of neurons. Rate-matched Fano Factor also exhibited very similar time course as the measure computed based on the entire sample of neurons (contrast Fig. 3B and 2B). The results indicate that increased firing rate accounts for a small part in the decrease of Fano Factor, however the variance of firing also declines after training, independent of firing rate.

### Effects of stimulus responsiveness and selectivity

**Figure 4:**
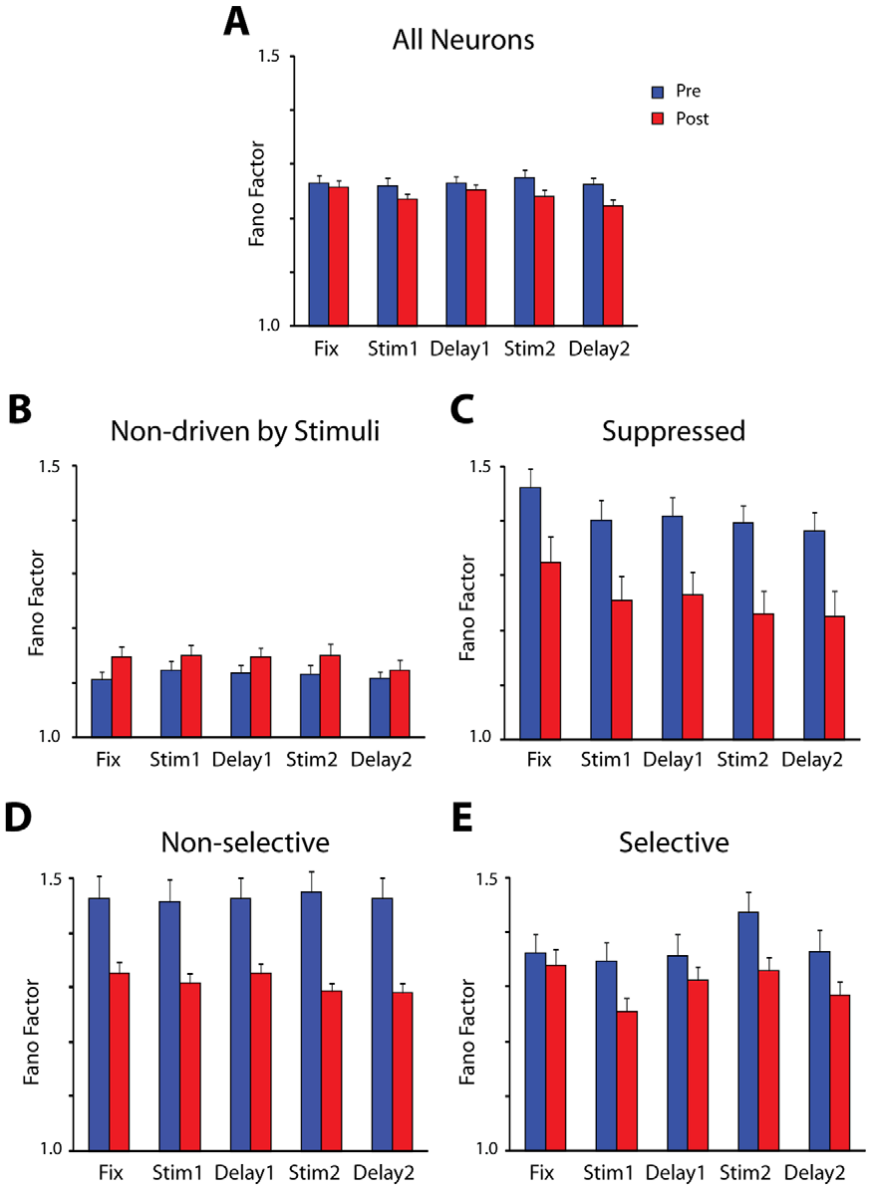
Average Fano Factor values for neurons with different properties. A) All recorded neurons, whether they responded to the task or not (N = 1279 pre; 1295 post-training). B) Neurons that did not respond to any aspect of the task (N = 583 pre; 498 post-training). C) Neurons that responded during a task period only with a suppressed response, and no excitatory response in any other period (N = 149 pre; 101 post-training). D) Neurons driven by the task but non-selective for the visual stimuli, in any task period (N = 313 pre; 288 post-training). E) Neurons selective for the visual stimuli (N = 175 pre; 207 post-training).

Analysis so far was based on neurons that responded to stimuli, the subset of neurons whose response properties we examined in detail before and after training [17], [18]. We refined our analysis of neuronal firing variability by examining the influence of stimulus responsiveness and selectivity, and in fact we observed differences for groups of neurons defined based on how they responded to stimuli, including the entire set of neurons sampled from the prefrontal cortex in an unbiased fashion (Fig. 4A). Prior to training, neurons that were not driven by any of the stimuli, exhibited the lowest Fano Factor values (Fig. 4B blue bars). The variability for this group of neurons was significantly lower (2-way ANOVA, and post hoc Tukey test, p<10^−5^) than neurons that were excited by any of the stimuli (Fig. 2A) or whose activity was exclusively suppressed by the stimuli (Fig. 4C, blue bars).

We also distinguished between neurons that were selective for the stimuli in at least one task period and estimated their variability in each period (whether a neuron was significantly selective for that period or not). We contrasted that, with neurons that were not selective in any period. Neurons that were not selective for the spatial stimuli (Fig. 4D) exhibited significantly higher (2-way ANOVA, p<0.001) levels of variability than the selective neurons (Fig. 4E). Differences in variability between neurons activated by different aspects of the task and selective to the stimuli presented may be attributed to task execution, however these results show that systematic differences between groups of neurons driven by stimuli are present even in the absence of training on a behavioral task.

After training, discharge variability declined when we considered our entire sample of neurons (Fig. 4A), or individual subgroups. One notable deviation from this pattern was that neurons that were not driven by the task exhibited significantly higher Fano Factor values after training (2-way ANOVA, p<0.01) compared to the equivalent group of neurons before training (Fig. 4B). Differences in the absolute level of Fano Factor between groups of neurons defined based on task responsiveness and stimulus selectivity continued to be present after training, with the group of neurons not driven by the stimuli exhibiting significantly lower levels of Fano Factor than neurons that were driven by the same stimuli (2-way ANOVA, p<10^−5^).

### Effects of Neuron Type

**Figure 5:**
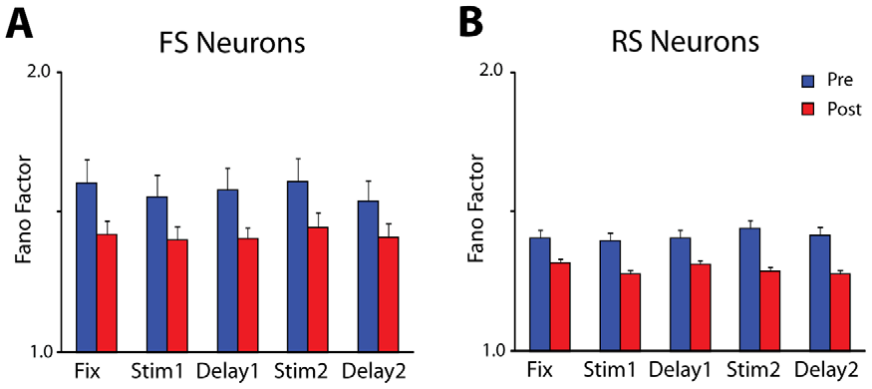
Fano Factors for FS and RS units. A) Average values of Fano Factor computed separately for FS units (putative interneurons, N = 52 pre; 45 post-training). B) Average values of Fano Factor for RS units (putative pyramidal neurons, N = 468 pre; 422 post-training).

We also determined variability separately for different types of neurons in terms of spike width properties. We distinguished between Fast Spiking (FS – putative interneurons) and Regular Spiking (RS – putative pyramidal neurons). Spike width measured extracellularly is not a precise indicator of neuron type, as large motor units in layer V of the motor and premotor cortex have been shown to display thin spikes [20]. It is notable however that we observed systematic differences between these types of neurons in our prefrontal recordings (which were obtained mostly from superficial layers). Prior to training (Fig. 5, blue bars), FS neurons exhibited a 17% higher average Fano Factor value over RS neurons, which represented a significant difference (2-way ANOVA, p<10^−5^). Both FS and RS neurons exhibited a significant overall decrease in Fano Factor values after training (3-way ANOVA, effect of training, p<0.001). This decrease however was proportionally larger for FS (9.1%) than RS neurons (3.4%), diminishing the contrast in variability between the FS and RS population after training.

### Variability during passive presentation

Considering that a number of changes in neuronal variability were observed after training, we wished to distinguish the effects caused by learning from those of executing the task itself. We therefore collected data from 139 neurons after training both during the execution of the task, and from blocks of trials when the monkey was only required to fixate while the same stimuli were presented passively. During the passive fixation blocks of trials, the choice targets were not displayed and the monkey was rewarded for maintaining fixation until the end of the second delay period, just as prior to training. Before collection began in the passive fixation condition, a series of trials with the stimulus appearing always at the same location were presented in order to condition the animals about the nature of the passive fixation condition, as described previously [18].

**Figure 6:**
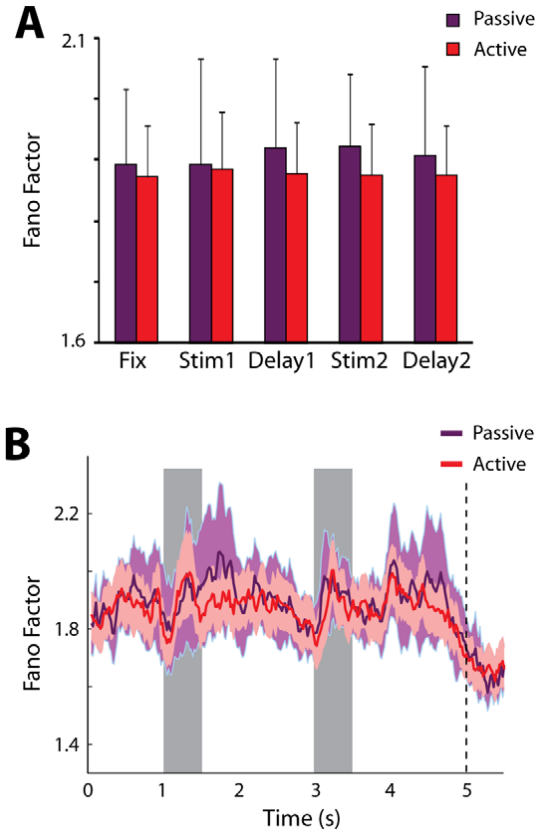
Variability during passive fixation after training. A) Average values of Fano Factor during the active execution of the Match/Nonmatch task and during passive presentation of the same stimuli, after training. The exact same neurons (N = 139) are compared in the two conditions. B). Time course of Fano Factor changes during active and passive presentation.

The Fano Factor of neuronal discharges exhibited an overall increase of 1.6% during the passive fixation condition compared to task execution condition (Fig. 6), however the difference did not reach statistical significance (2-way ANOVA, p>0.6). We saw similar changes in Fano Factor values across task epochs (Fig. 6A). Importantly, the time course of Fano Factor during the second delay period was virtually identical in the two conditions, even though no judgment was required at the end of the period for the passive fixation condition (Fig. 6B). The results indicate that executing the task further decreases neuronal variability, though the effects of training and learning to perform the task shape neuronal variability to a greater extent.

### Effects of Match/Nonmatch judgment

**Figure 7:**
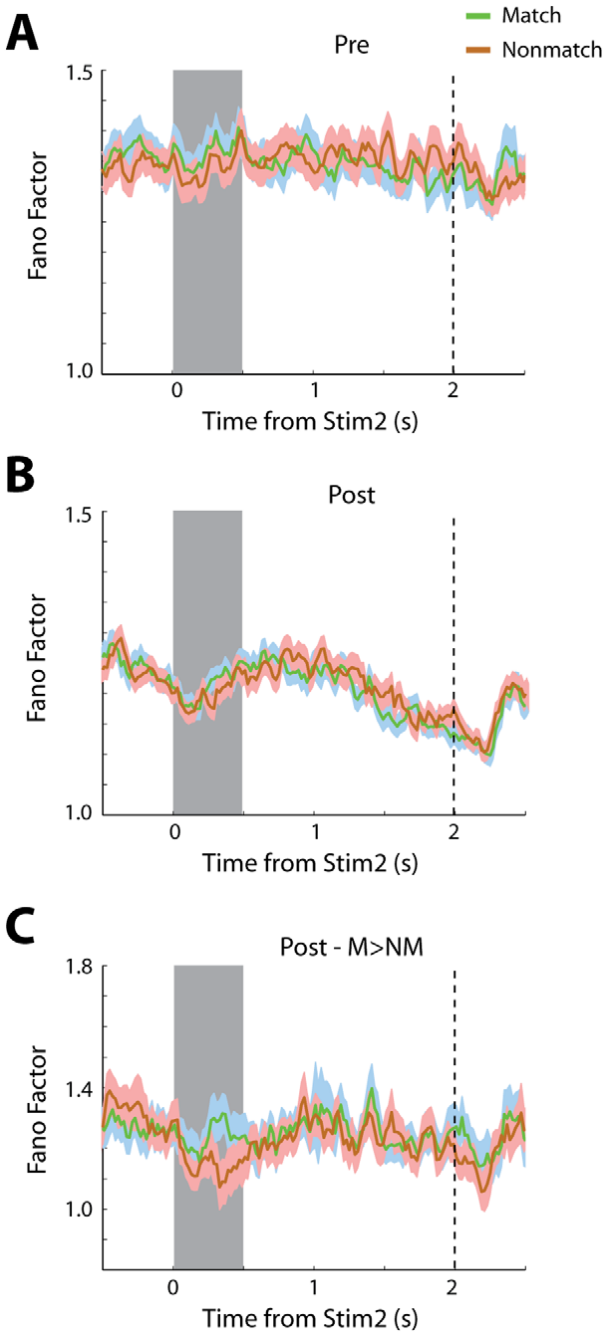
Variability for match and nonmatch stimuli. A) Time course of Fano Factor values from all neurons recorded prior to training plotted separated by trials that contained Match and Nonmatch stimuli, after training. B). Time course of Fano Factor values for all neurons recorded after training. C) Time course of Fano Factor values for neurons recorded after training with significant preference for a match over a nonmatch stimulus (N = 53).

To further examine the effect of imposing a decision on neuronal variability we distinguished between trials where the second stimulus matched the first or not. We have recently identified neurons in this context of this task, whose responses to the identical stimulus are significantly different when it is preceded by the same stimulus and therefore constitutes a match, and when it is preceded by a different stimulus and constitutes a nonmatch [21]. Across the entire population, there was no significant difference in discharge variability in trials that contained match stimuli, and those that contained nonmatch ones. This was true, both prior to training (Fig. 7A), and after (Fig. 7B) training (2-way ANOVA test, p>0.8 in either case). We did however detect a significant difference in variability specifically for those neurons that also exhibited a significant difference in firing rate, preferring the match stimulus over the nonmatch (Fig. 7C). For this population of neurons, variability in match trials was significantly lower than nonmatch ones, during the time of the second stimulus presentation (paired t-test, p<0.05). No significant difference was seen for the equivalent population prior to training, or for neurons that preferred nonmatch over match stimuli after training (data not shown).

## Discussion

Our study quantified the variability of neuronal discharges in naïve animals and determined the effects of learning and performing a working memory task. We found that overall, Fano Factor values decreased after training. Systematic differences existed between populations of neurons characterized by the responsiveness and selectivity to stimuli used in the task but these were present both before and after training. Presenting stimuli under fixation conditions, after the animals had learnt to perform the working memory task revealed that distinct changes were present after training even when the animals were not executing a task.

### Influence of cognitive functions on neuronal variability

The variability of neuronal responses is well documented; firing rate of cortical neurons is considerably variable from trial to trial even when the identical stimulus is presented under controlled experimental conditions [22], [23], [24], [25], [26]. This has been traditionally thought to be the inescapable result of noisy computations performed by biological circuits. More recently, it has been demonstrated that variability of neuronal responses elicited by stimuli decreases when attention is directed to them [11], is similarly reduced during the time interval of the presentation of stimuli [12], and increases during the course of decision making involving multiple options [14]. It has been recognized therefore that cognitive factors affect the properties of neuronal firing during the execution of behavioral tasks [10]. How performing a task itself affects neuronal variability has not been addressed so far. One possible way to discount task and attention effects is to present stimuli under passive fixation conditions, requiring no action of the subject [19]. An inherent problem with such a strategy however, is that it is impossible to ascertain whether the subject is still mentally performing the task to some extent. Instead, we relied on comparison of neuronal responses from a large sample of neurons in the same animals during execution of the spatial working memory task and before they had been trained to perform the task at all. Our comparison involved the presentation of the same stimuli and with the exact same timing, allowing us to minimize differences between the two sets of recordings. Our approach does have some limitations, as in the pre-training condition the animals may still have learned that the stimulus sequence was informative about the trial progress and they may have anticipated the approaching reward. However these factors were identical after training and the changes we report may only be accounted by learning to perform and executing the task.

### Effects of Training

Neuronal discharge variability in our study decreased after training. The magnitude of the changes we report (7.5% for neurons responding to the stimuli) was comparable to effects previously described between stimulus conditions in trained animals [12], [27], which demonstrated the effect of cognitive factors on neuronal firing in the first place. Our results replicated the decreases in Fano Factor value during the stimulus presentation, in agreement with reports from multiple other brain areas [12]. This stimulus-related decrease was present both before and after training to perform the task. This finding indicates that learning to perform a task reduces neuronal variability of neuronal responses throughout the trial and interacts in an additive way with the effect of the stimulus presentation.

Changes in variability were not uniform but affected differently populations of neurons with different properties in the task. After training, we observed large decreases for neurons that were driven by the stimuli (Fig. 4B) but actually an increase in Fano Factor values for neurons that were suppressed by the stimuli, compared to equivalent neurons recorded before training (Fig. 4C). Variability between groups of neurons responding to different aspects of the task is commonly attributed to task execution [19]. We show that these differences are in fact present in the absence of task execution, even prior to training in any task.

Decreased levels of neuronal variability have been previously suggested as signatures of motor preparation [13] and our results add performance of a working memory task to the list of factors that modulate variability. From an information decoding standpoint, decreased variability has been shown to improve the efficiency in neural coding and increase the information encoded in neuronal firing [28]. Our study suggests that performing a working memory task that requires manipulation of the stimulus information can improve information decoding through decreased variability.

## Materials and Methods

### Ethics Statement

All animal experiments were performed in compliance with the guidelines set forth by the National Institutes of Health, as reviewed and approved by the Wake Forest University Institutional Animal Care and Use Committee. Behavioral training was accomplished via fluid regulation; animals received the same minimum amount of fluids every day, regardless of whether they performed the task or not. All surgeries were performed using aseptic techniques in an approved surgical suite and under appropriate anesthesia. Approved analgesics were delivered post-operatively, under the guidance of veterinary staff. Animals were provided with environmental enrichment designed by the institutional environmental enrichment coordinator.

### Surgery and Neurophysiology

Three male, rhesus monkeys (*Macaca mulatta*) weighing 5–12 kg were used in this study. Neuronal recordings were obtained from the lateral prefrontal cortex of the monkeys (Fig. 1A) before and after training in a working memory task, as previously described in more detail [18]. Recordings were obtained with multiple (up to 8), independently movable Tungsten microelectrodes that were advanced into the cortex with a microdrive system (EPS drive, Alpha-Omega Engineering, Nazareth, Israel). We used glass-coated 250 μm diameter electrodes with an impedance of 1 MΩ at 1 kHz (Alpha-Omega Engineering, Nazareth, Israel) and epoxylite-coated 125 μm diameter electrodes with an impedance of 4 MΩ at 1 KHz (FHC Bowdoin, ME). Neural signals were band-pass filtered between 500 Hz and 8 kHz and recorded with a modular data acquisition system at a 25 μs sampling resolution (APM system, FHC, Bowdoin, ME). Any neurons isolated during advancement of electrodes were sampled with no effort to pre-screen them based on functional properties. Data analysis was performed using the MATLAB computational environment (Mathworks, Natick, MA).

#### Behavioral Task

Neuronal data were compared at two stages of recordings. In the first stage (prior to working memory training), the monkeys were only required to maintain fixation while stimuli were presented on a screen (Fig. 1B). The stimulus appeared for 0.5 s and was followed by a “delay period” that lasted for 1.5 s. After the delay period, a second white square appeared either at the same location, or a different (typically diametric) location for 0.5 s. This was followed by a second 1.5 s delay period. The stimuli analyzed here were 2° white squares that appeared randomly at any of 9 locations arranged on a 3×3 grid; the distance between adjacent stimulus locations was 10° (Fig. 1C). All stimuli were presented using in-house software [29]. After recordings were obtained at this stage, the animals were trained to perform a spatial working memory task. The stimuli and timing of presentation were identical before and after working memory training, allowing us to compare neuronal properties between stages. A second set of recordings was then obtained after training had been completed. Average behavioral performance in the sessions analyzed here was 89%. Data from correct trials only are presented in this paper. Typically 20 trials were recorded for each cue stimulus condition (180 trials from each neuron studied).

#### Neuron Analysis and Classification

Action-potential waveforms recorded from all neurons we encountered (which were sampled in an unbiased fashion) were sorted into separate units using an automated cluster analysis method based on the KlustaKwik algorithm [30]. A neuron's spike width was determined by calculating the distance between the two troughs of the average waveform. We distinguished between Fast Spiking (FS – putative interneurons) and Regular Spiking (RS – putative pyramidal) neurons based on previous analysis [6], [18]; units were classified as FS if they exhibited as spike width of ≤550 μs and RS if they exhibited a spike width of ≥575 μs. Spike width measured extracellularly is not a precise indicator of neuron type; it has been recently shown that pyramidal neurons in the motor and premotor cortex that project to the spinal cord display thin spikes [20]. It is notable however that we observed systematic differences between these types of neurons in our prefrontal recordings (which were obtained mostly from superficial layers), and errors in classification are likely to dilute any difference between the populations of pyramidal neurons and interneurons.

Firing rate of each neuron was subsequently determined for each of the task epochs. We identified neurons that responded to the visual stimuli, evidenced by significantly elevated firing rate in the 0.5 s interval of a stimulus presentation, compared to the 1 s interval of fixation (paired t-test, p<0.05). Neurons with significantly elevated or reduced activity in other task epochs were similarly identified. Firing rate comparisons always included the entire interval of the stimulus or delay window (0.5 s for the stimulus and 1.5 s of delay period). The functional properties of neurons in this dataset have been described in detail elsewhere [17], [18]. In addition, we examine here variability of neurons that did not respond to any of the stimuli, based on these statistical criteria.

#### Fano Factor

The Fano Factor of a neuron's discharge rate (defined as the variance divided by the mean) was estimated in different task periods. We relied on the method of Churchland and colleagues [12], using the algorithm developed by these authors and made available at: http://www.stanford.edu/~shenoy/GroupCodePacks.htm. Data for each neuron and stimulus location were initially treated separately. Spike counts were computed in a 100-ms sliding window moving in 20-ms steps. The method computes the variance and mean of the spike count across trials and performs a regression of the variance to the mean. This slope of this regression represents the Fano Factor reported here.

To discount the effect of firing rate on Fano Factor, data before and after training were rate-matched with the following procedure, similar to that used previously [12]. Firing rates from all stimulus conditions were first averaged together for each neuron. Neurons were then grouped in 0.2 sp/s bins based on firing rate during the stimulus period. In each bin, equal numbers of neurons recorded before and after training were randomly selected for analysis. The procedure produced two samples with equal numbers of neurons, matched for firing rate during the stimulus period. Matching based on other task periods was also performed, with similar results.

## References

[1] Shadlen MN, Newsome WT (1998). The variable discharge of cortical neurons: implications for connectivity, computation, and information coding.. Journal of Neuroscience.

[2] Faisal AA, Selen LP, Wolpert DM (2008). Noise in the nervous system.. Nat Rev Neurosci.

[3] Gawne TJ, Richmond BJ (1993). How independent are the messages carried by adjacent inferior temporal cortical neurons?. Journal of Neuroscience.

[4] Lee D, Port NL, Kruse W, Georgopoulos AP (1998). Variability and correlated noise in the discharge of neurons in motor and parietal areas of the primate cortex.. Journal of Neuroscience.

[5] Maynard EM, Hatsopoulos NG, Ojakangas CL, Acuna BD, Sanes JN (1999). Neuronal interactions improve cortical population coding of movement direction.. Journal of Neuroscience.

[6] Constantinidis C, Goldman-Rakic PS (2002). Correlated discharges among putative pyramidal neurons and interneurons in the primate prefrontal cortex.. Journal of Neurophysiology.

[7] Kohn A, Zandvakili A, Smith MA (2009). Correlations and brain states: from electrophysiology to functional imaging.. Curr Opin Neurobiol.

[8] Ecker AS, Berens P, Keliris GA, Bethge M, Logothetis NK (2010). Decorrelated neuronal firing in cortical microcircuits.. Science.

[9] Averbeck BB, Latham PE, Pouget A (2006). Neural correlations, population coding and computation.. Nat Rev Neurosci.

[10] Cohen MR, Kohn A (2011). Measuring and interpreting neuronal correlations.. Nat Neurosci.

[11] Mitchell JF, Sundberg KA, Reynolds JH (2007). Differential attention-dependent response modulation across cell classes in macaque visual area V4.. Neuron.

[12] Churchland MM, Yu BM, Cunningham JP, Sugrue LP, Cohen MR (2010). Stimulus onset quenches neural variability: a widespread cortical phenomenon.. Nat Neurosci.

[13] Churchland MM, Afshar A, Shenoy KV (2006). A central source of movement variability.. Neuron.

[14] Churchland AK, Kiani R, Chaudhuri R, Wang XJ, Pouget A (2011). Variance as a signature of neural computations during decision making.. Neuron.

[15] Cohen MR, Newsome WT (2008). Context-dependent changes in functional circuitry in visual area MT.. Neuron.

[16] Cohen MR, Newsome WT (2009). Estimates of the contribution of single neurons to perception depend on timescale and noise correlation.. J Neurosci.

[17] Meyer T, Qi XL, Stanford TR, Constantinidis C (2011). Stimulus selectivity in dorsal and ventral prefrontal cortex after training in working memory tasks.. J Neurosci.

[18] Qi XL, Meyer T, Stanford TR, Constantinidis C (2011). Changes in Prefrontal Neuronal Activity after Learning to Perform a Spatial Working Memory Task.. Cerebral Cortex.

[19] Hussar C, Pasternak T (2010). Trial-to-trial variability of the prefrontal neurons reveals the nature of their engagement in a motion discrimination task.. Proc Natl Acad Sci U S A.

[20] Vigneswaran G, Kraskov A, Lemon RN (2011). Large identified pyramidal cells in macaque motor and premotor cortex exhibit “thin spikes”: implications for cell type classification.. J Neurosci.

[21] Qi XL, Meyer T, Stanford TR, Constantinidis C (2012). Neural correlates of a decision variable before learning to perform a Match/Nonmatch task.. Journal of Neuroscience.

[22] Tolhurst DJ, Movshon JA, Dean AF (1983). The statistical reliability of signals in single neurons in cat and monkey visual cortex.. Vision Research.

[23] Dean AF (1981). The variability of discharge of simple cells in the cat striate cortex.. Experimental Brain Research.

[24] Vogels R, Spileers W, Orban GA (1989). The response variability of striate cortical neurons in the behaving monkey.. Exp Brain Res.

[25] McAdams CJ, Maunsell JH (1999). Effects of attention on the reliability of individual neurons in monkey visual cortex.. Neuron.

[26] Softky WR, Koch C (1993). The highly irregular firing of cortical cells is inconsistent with temporal integration of random EPSPs.. Journal of Neuroscience.

[27] Cohen MR, Maunsell JH (2009). Attention improves performance primarily by reducing interneuronal correlations.. Nat Neurosci.

[28] Oram MW (2010). Visual stimulation decorrelates neuronal activity.. J Neurophysiol.

[29] Meyer T, Constantinidis C (2005). A software solution for the control of visual behavioral experimentation.. J Neurosci Methods.

[30] Harris KD, Henze DA, Csicsvari J, Hirase H, Buzsaki G (2000). Accuracy of tetrode spike separation as determined by simultaneous intracellular and extracellular measurements.. J Neurophysiol.

